# Metabolic syndrome and dietary components are associated with coronary artery disease risk score in free-living adults: a cross-sectional study

**DOI:** 10.1186/1758-5996-3-7

**Published:** 2011-05-09

**Authors:** Mauro Massao Takahashi, Erick Prado de Oliveira, Ana Lygia Rochitti de Carvalho, Lidiane Affonso de Souza Dantas, Franz Homero Paganini Burini, Kátia Cristina Portero-McLellan, Roberto Carlos Burini

**Affiliations:** 1 Centre for Nutritional and Physical Exercise Metabolism, UNESP School of Medicine, Public Health Department, Botucatu City, São Paulo State; Brazil; 2 Pathology graduate student, UNESP School of Medicine - UNESP, Botucatu City, São Paulo State; Brazil; 3 São Paulo University (USP), São Paulo, Brazil

## Abstract

**Background:**

Coronary artery disease (CAD) is among the main causes of death in developed countries, and diet and lifestyle can influence CAD incidence.

**Objective:**

To evaluate the association of coronary artery disease risk score with dietary, anthropometric and biochemical components in adults clinically selected for a lifestyle modification program.

**Methods:**

362 adults (96 men, 266 women, 53.9 ± 9.4 years) fulfilled the inclusion criteria by presenting all the required data. The Framingham score was calculated and the IV Brazilian Guideline on Dyslipidemia and Prevention of Atherosclerosis was adopted for classification of the CAD risks. Anthropometric assessments included waist circumference (WC), body fat and calculated BMI (kg/m^2^) and muscle-mass index (MMI kg/m^2^). Dietary intake was estimated through 24 h dietary recall. Fasting blood was used for biochemical analysis. Metabolic Syndrome (MS) was diagnosed using NCEP-ATPIII (2001) criteria. Logistic regression was used to determine the odds of CAD risks according to the altered components of MS, dietary, anthropometric, and biochemical components.

**Results:**

For a sample with a BMI 28.5 ± 5.0 kg/m^2 ^the association with lower risk (<10% CAD) were lower age (<60 years old), and plasma values of uric acid. The presence of MS within low, intermediary, and high CAD risk categories was 30.8%, 55.5%, and 69.8%, respectively. The independent risk factors associated with CAD risk score was MS and uric acid, and the protective factors were recommended intake of saturated fat and fiber and muscle mass index.

**Conclusion:**

Recommended intake of saturated fat and dietary fiber, together with proper muscle mass, are inversely associated with CAD risk score. On the other hand, the presence of MS and high plasma uric acid are associated with CAD risk score.

## Background

Coronary artery disease (CAD) is among the main causes of death in developed countries, and it currently occurs with greater frequency in developing countries, especially among the elderly [1,2]. The Framingham score was developed to estimate the CAD risk over 10 years and it was validated for people aged up to 75 years [3,4]. The PRINCEPS (*Identification Program of Cardiovascular Risk Level and Increase in Lipid Parameters*) study conducted in Spain between 2004 and 2005 with more than 26,500 individuals of both genders above 45 years of age, observed that established CAD prevalence or CAD risk of 36.9% (n = 9829) [5]. This same study found a prevalence of 34.9% (n = 9292) of individuals with CAD risk higher than 20% in 10 years [5].

CAD risk can be calculated from risk factors and the presence of clinical signs and biochemical abnormalities [6]. The Framingham risk score is often used as an initial evaluation parameter of CAD risk in individuals with countless risk factors, including those with Metabolic Syndrome (MS) [7].

The MS is a multiple risk factor for cardiovascular disease and is characterized by increased waist circumference, raised triglycerides, reduced HDL cholesterol, elevated blood pressure, and raised plasma glucose [8,9]. From these, reduced HDL-c and elevated blood pressure are common components of both CAD risk and MS.

Diet and lifestyle can influence CAD incidence [10]. A Mediterranean diet, rich in plant foods in combination with nonsmoking, moderate alcohol consumption, and daily physical activity is associated with a significantly lower mortality rate of CAD and all cause mortality [11]. Some factors related to MS such as dyslipidemia, hyperglycemia, hypertension, obesity and other risk factors like low levels of physical activity and smoking [12] have already been well established as CAD risk factors. However, most Brazilian studies investigate the association of factors related to lifestyle changes with CAD risk factors only individually [13-16] (e.g., hypertension, diabetes, hypercholesterolemia); but no national study investigated the association between CAD risk (Framingham score) and its direct relationship with dietary components, biochemical and body composition [17,18].

The aim of this study was to evaluate the association of CAD risk score with anthropometric, biochemical and dietary factors in adults with or without MS who were clinically selected for a lifestyle modification program.

## Methods

### Individuals

A descriptive cross-sectional study was conducted in a subgroup of participants clinically screened for a lifestyle modification program "*Mexa-se Pró-Saúde *[Move for Health]", from 2004 to 2007. This program is offered to patients with non-communicable chronic diseases and consists of regular physical exercise and nutritional counseling. The Metabolism, Exercise and Nutrition Center (CeMENutri), conducts this program since 1992, in Botucatu. Botucatu is a city located in the center of Sao Paulo State, about 230 km west of the capital city and has a population of 121,274 in habitants [19]. The inclusion criteria for participants were individuals over the age of 35, of both genders, with at least one of the MS components and/or comorbities, and without metabolic or motor disabilities that would limit physical exercise.

A total of 658 individuals were attended at the program during this period. From those individuals, 362 individuals had CHD data and were studied. The study included 96 men and 266 women, with age of 53.9 ± 9.4 and BMI of 28.5 ± 5.0 kg/m^2^. Besides gender the individuals were also distributed by age considering 60 years the cutoff for elderly. All had signed a term of informed consent, and the project was approved by the Research Ethics Committee (CEP protocol 3271-2009) of the Botucatu Medical School (FMB), São Paulo State University (UNESP, Brazil).

### Clinical Evaluation of Arterial Blood Pressure

Systolic and diastolic arterial blood pressure was evaluated with the individual in the seated position according to the procedures described by the V Brazilian Guidelines on Arterial Hypertension [20]. Values of systolic blood pressure ≥ 130 mm Hg and/or diastolic blood pressure ≥85 mm Hg were considered abnormal.

### Biochemical Analyses

Blood samples were collected after overnight fasting 10 to 12 hours using a vacuum venous puncture. The individuals were previously instructed to not perform vigorous physical exercise 24 hours and/or not drink alcohol 72 hours before collection. Laboratory analyses of lipid parameters (total cholesterol, fractions and triglycerides), glucose, uric acid, urea, γ-glutamyl transferase (γ-GT), C-reactive protein (CRP), albumin and total proteins were performed within 4 hours after blood collection using the Dry Chemical method (Vitros^® ^system, Johnson & Johnson), at the Clinical Analyses Laboratory of the Teaching Hospital (School of Medicine UNESP) in Botucatu/SP. The non-high density lipoprotein cholesterol (nHDL-c) values were obtained finding the difference between total cholesterol (TC) and high density lipoprotein cholesterol (HDL-c). Concentrations of triglycerides (TG) >150 mg/dL [21], TC >200 mg/dL [22], HDL-c <40 mg/dL for men and <50 mg/dL for women [21], glycemia ≥100 mg/dL [23] and uric acid (higher quartile) ♂ > 6,5 mg/dL and ♀ > 5 mg/dL) were considered abnormal [24,25].

### Risk of Coronary Artery Disease (CAD)

In order to estimate the Framingham score and calculate CAD risk over 10 years, the classification from the IV Brazilian Guideline on Dyslipidemia and the Prevention of Arteriosclerosis [22] was adopted. Individuals who reported diabetes mellitus (type 1 or 2) through clinical protocol were included in phase 1 of risk stratification, which is considered a clinical manifestation equivalent to arteriosclerotic disease. Thus, the population with diabetes has a 20% greater risk of presenting cardiovascular events in 10 years. Phase 2 of the stratification considers the risk by estimating Framingham scores, where after adding the points obtained for each variable (gender, age, systolic blood pressure, TC, HDL-C and smoking), the absolute risk percentage in 10 years was calculated, which can be classified as low risk (<10%), intermediate risk (10 to 20%) and high risk (>20%).

### Body Composition

Body weight was measured on a platform anthropometric scale (Filizola^®^) with maximum capacity of 150 kg and precision of 0.1 kg. Height was determined using a portable Seca^® ^stadiometer, with a precision of 0.1 cm [26]. After body weight and height was evaluated, BMI was calculated (weight/height (m)^2^) and classified [27].

Waist circumference (WC) was measured at the point midway between the last rib and the iliac crest. All measurements used the *Sanny*^® ^steel anthropometric tape measure. Values in excess of 102 cm for men and 88 cm for women [25] were considered elevated.

Bioelectric impedance (Biodynamics^®^, model 450, USA) was used to determine the percentage of body fat (%BF) and muscle mass (kg). Segal *et al *(1988) equation was used to calculated the %BF [28]. Values ranging between 15 and 25% and 20 and 35% for men and women, respectively, were considered normal for (%BF) [29]. The percentage of muscle mass (%MM) was obtained using the Janssen *et al.*, (2000) equation [30] and the muscle mass index (MMI) was calculated as MM (kg)/height^2^. Individuals were classified as sarcopenic if their values were below 10.75 kg/m^2 ^and 6.75 kg/m^2 ^for men and women, respectively [31].

### Metabolic Syndrome

Diagnosis of Metabolic Syndrome was made according to the criteria of NCEP-ATP III [24,25]. The 5 components used were plasma levels of triglyceride, HDL-C and, fasting plasma glucose, systolic and diastolic blood pressure and WC measurements. Metabolic syndrome was diagnosed when 3 or more of these components were abnormal.

### Dietary Assessment

The 24 h dietary recall was used to assess food intake [32]. Dietary data obtained in homemade measurements were converted into grams and milliliters to permit chemical analysis of food intake. The centesimal composition of foods present in the records was calculated using NutWin^® ^(2002) software, version 1.5. Foods not found in the software were added from diverse composition tables and food labels [33,34]. Diet quality was evaluated using the Adapted Healthy Eating Index (HEI) [35] and evaluated groups were based on portions recommended by the Adapted Food Pyramid [36].

### Statistical Analysis

The descriptive characteristics were presented as mean and standard deviation, applying the ANOVA and Tukey test to compare means. Regression models negative binomial were adjusted to characterize portions intake. The Spearman correlation was used to evaluate the correlation of demographic, anthropometric, dietary, biochemical, systolic and diastolic blood pressure and MS components with the Framingham risk score. Logistic regression was used to determine the probability of high CAD risk score (low+moderate vs high) according to dietary (adjusted for TCV + BMI), and anthropometric components, MS, CRP and uric acid concentration (adjusted for BMI). A value of p < 0.05 was adopted as significant. The SAS program, version 9.1.3, was used for data analysis.

## Results

Table 1 shows the distribution of the variables' average values according to the seriousness of CAD risk score. Individuals with the lower risk had the youngest age, lowest waist circumference, lowest legume intake, lowest triglyceridemia, uricemia and diastolic blood pressure values, and highest concentrations of HDL-C. Individuals with the intermediate risk had the highest MMI, total cholesterolemia, LDL-C and nHDL-C values, and lowest CRP values. Individuals with the higher CAD risk score had the highest energy intake and highest plasma values for glucose and urea. The presence of MS within low, intermediate and high CAD risk score categories was 30.8%, 55.5% and 69.8%, respectively (data not shown).

**Table 1:** Demographic, anthropometric, dietary and biochemical characteristics of the sample according to CAD risk score classification in free-living adults

	Low Risk	Intermediate Risk	High Risk
Age (years)	52.1 ± 8.9^a^	59.4 ± 8.1^b^	56.6 ± 9.9^b^
BMI (kg/m2)	28.3 ± 5.1^a^	28.8 ± 4.6^a^	29.1 ± 5.0^a^
% Body Fat	32.4 ± 8.8^a^	30.3 ± 6.9^a^	31.9 ± 7.6^a^
Waist circumference (cm)	94.6 ± 12.3^a^	100.6 ± 14.0^b^	100.0 ± 13.2^b^
Muscle Mass Index (kg/m^2^)	8.1 ± 1.4^a^	9.3 ± 1.7^b^	8.4 ± 1.5^a^
Total energy intake (kcal)	1538 ± 484^a^	1600 ± 470^a^	1920 ± 912^b^
HEI (points)	84.3 ± 13.6^a^	83.0 ± 11.6^a^	83.6 ± 16.6^a^
Carbohydrates (% of energy)	51.6 ± 8.8^a^	54.3 ± 9.6^a^	52.5 ± 7.9^a^
Proteins (% of energy)	18.7 ± 5.1^a^	19.9 ± 4.3^a^	18.2 ± 5.8^a^
Proteins (g/kg weight)	1.0 ± 0.4^a^	0.9 ± 0.3^a^	1.0 ± 0.4^a^
Lipids (% of energy)	29.9 ± 8.7^a^	27.2 ± 9.5^a^	29.1 ± 6.1^a^
SFA (% of energy)	8.3 ± 3.8^a^	6.2 ± 2.3^a^	7.2 ± 3.3^a^
MUFA (% of energy)	9.0 ± 3.7^a^	8.3 ± 3.8^a^	8.6 ± 2.5^a^
PUFA (% of energy)	6.9 ± 3.7^a^	7.8 ± 4.7^a^	7.9 ± 3.0^a^
Cholesterol (mg)	162.6 ± 103.6^a^	147.7 ± 70.5^a^	206.0 ± 140.6^a^
Fibers (g)	13.2 ± 7.5^a^	16.0 ± 8.8^a^	16.1 ± 9.0^a^
Cereal (portions)	3.3 ± 1.5^a^	3.5 ± 1.0^a^	3.8 ± 1.4^a^
Fruit (portions)	3.3 ± 3.0^a^	3.8 ± 3.5^a^	2.7 ± 3.0^a^
Vegetables (portions)	2.4 ± 2.3^a^	3.3 ± 2.9^a^	3.2 ± 4.2^a^
Legumes (portions)	0.8 ± 1.1^a^	1.5 ± 1.7^b^	1.5 ± 2.5^b^
Dairy Products (portions)	1.8 ± 1.3^a^	1.5 ± 1.1^a^	2.0 ± 1.6^a^
Meat (portions)	1.8 ± 1.3^a^	1.8 ± 1.2^a^	1.8 ± 1.0^a^
Sugar (portions)	1.7 ± 1.8^a^	1.9 ± 2.5^a^	1.8 ± 3.2^a^
Oil (portions)	2.4 ± 2.3^a^	2.2 ± 1.1^a^	3.1 ± 2.1^a^
Variety (item)	13.5 ± 3.7^a^	14.2 ± 3.4^a^	13.7 ± 4.4^a^
Total cholesterol (mg/dL)	203.8 ± 35.4^a^	225.7 ± 49.4^b^	201.3 ± 42.7^a^
Glucose (mg/dL)	91.7 ± 18.2^a^	95.4 ± 14.1^a^	129.4 ± 50.2^b^
Triglycerides (mg/dL)	140.8 ± 61.1^a^	169.8 ± 70.1^b^	172.7 ± 69.3^b^
HDL-C (mg/dL)	52.7 ± 12.4^a^	44.8 ± 9.3^b^	45.7 ± 12.2^b^
LDL-C (mg/dL)	122.9 ± 31.6^a^	146.8 ± 46.0^b^	121.0 ± 41.3^a^
Urea ((mg/dL)	31.9 ± 9.6^a^	33.4 ± 7.2^a^	37.4 ± 18.7^b^
Uric acid (mg/dL)	4.8 ± 1.3^a^	5.9 ± 1.6^b^	5.6 ± 1.5^b^
γ-GT (mg/dL)	31.8 ± 22.4^a^	36.1 ± 18.9^a^	32.1 ± 19.1^a^
nHDL-C (mg/dL)	151.1 ± 35.4^a^	180.8 ± 46.6^b^	155.6 ± 44.7^a^
CRP (mg/dL)	0.52 ± 0.57^a^	0.29 ± 0.21^b^	0.65 ± 0.96^a^
SBP (mm/Hg)	124.6 ± 15.6^a^	140.1 ± 16.8^b^	131.4 ± 16.0^c^
DBP (mm/Hg)	79.6 ± 8.9^a^	84.1 ± 8.1^b^	81.4 ± 6.0^b^

*CAD: coronary artery disease; BMI: body mass index; HEI: healthy eating index; SFA: saturated fatty acid; MUFA: monounsaturated fat acid; PUFA: polyunsaturated fatty acid; CAD: coronary artery disease; HDL-C: high-density lipoprotein; LDL-C: low-density lipoprotein; nHDL-C: non high-density lipoprotein; CRP: C-reactive protein; SBP: systolic blood pressure; DBP: diastolic blood pressure; γ-GT: γ-glutamyl transferase. Different letters indicate significant differences (p < 0.05)*.

Table 2 shows the significant and stronger (r > 0.3) correlations of demographic, anthropometric, dietary and biochemical data with CAD risk score. Positive correlations were observed with age, % energy from protein, glucose, uric acid, SBP, DBP and number of MS components. The only negative correlation was with HDL-c.

**Table 2:** Significant correlation of demographic, anthropometric, dietary and biochemical data with CAD risk score (p < 0.05)

	CAD Risk Score	p Value
Age	0.420	<0.0001
% energy from Protein	0.309	0.012
Glucose	0.374	<0.0001
HDL-C	-0.323	<0.0001
Uric acid	0.370	<0.0001
SBP	0.461	<0.0001
DBP	0.358	<0.0001
MS (number of components)	0.453	<0.0001

CAD: coronary artery disease; HDL-C: high-density lipoprotein; LDL-C: low-density lipoprotein; nHDL-C: non high-density lipoprotein; SBP: systolic blood pressure; DBP: diastolic blood pressure; MS: metabolic syndrome

Odds Ratios for CAD risk score can be found in Table 3, high plasma uric acid and presence of metabolic syndrome were risk factors and muscle mass index a protective factor. Furthermore, recommended intake of saturated fat (<10% TCV) and dietary fiber (>20g/day) [21] acted as protective dietary factors for CAD risk score, even after adjustments for BMI and TCV.

**Table 3:** Odds ratio for CAD risk score according to anthropometry, diet, MS, CRP and uric acid concentrations

	Model 1	Model 2
BMI (≥25 vs <25 kg/m^2^)	1.540 (0.900-2.635)	-
Waist circumference¹	1.492 (0.939-2.372)	1.480 (0.828-2.647)
Muscle Mass IndexMI^2^	0.333 (0.140-0.794)*	0.297 (0.110-0.799)*
% Body fat^3^	1.437 (0.861-2.398)	1.394 (0.667-2.917)
MS^4^	3.906 (2.450-6.250)*	4.276 (2.581-7.083)*
CRP (≥1.0 vs <1.0 mg/dL)	0.580 (0.220-1.531)	0.499 (0.180-1.380)
Uric acid^5^	3.856 (1,190-12,493)*	3.552 (1.081-11.668)*
Saturated fat acids (<10% vs ≥ 10% TCV)	0.301 (0.121-0.752)*	0.269 (0.098-0.378)*
Dietary fiber (≥ 20 vs < 20g/d)	0.309 (0.151-0.633)*	0.297 (0.132-0.668)*

Model 1: crude

Model 2: adjusted for BMI (for all variables) + TCV (for dietary variables)

95% CI; *p < 0.05; CAD: coronary artery disease; MS: metabolic syndrome; CRP: C-reactive protein; BMI: body mass index,; ¹: cutoff points ♂ (>102 vs ≤102 cm) and ♀ (>88 vs ≤88 cm); ^*2*^*: cutoff points ♂ (≥10.75 vs <10.75 kg/m*^*2*^*) and ♀ (≥6.75 vs <6.75 kg/m*^*2*^*); *^*3*^*: cutoff points ♂ (≥25 vs <25%) and ♀ (≥35 vs <35%); *^*4*^*: presence vs absence; *^*5*^*: cutoff points (♂ > 6,5 mg/dL and ♀ > 5 mg/dL)*

In general, besides the variables used to calculate CAD risk score, muscle mass and recommended intake of saturated fat and fiber were associated as protective factors, and the presence of metabolic syndrome was associated as risk factor.

## Discussion

As expected [9], in this study, CAD risk score increased with age and was related to its diagnostic elements, SBP, TC (nHDL-C) and HDL-C. Furthermore, a strong positive influence of MS and its components (WC, glucose and TG) was observed in CAD risk score. From these, blood pressure and HDL-c are less valid due to the fact they are both CAD risk and MS diagnostic elements.

From the logistic regression analyses, individuals with MS presented a fourfold greater probability of high CAD risk score. The same was observed by Wannamethee et al., (2005) [37], where men with MS presented a relatively significant risk (RR 1.64; 95% CI; 1.26-2.06) for developing CAD compared to individuals without MS.

The association between MS and CAD risk found in this study was similar the one observed in studies conducted in the United States [38,39] and Europe [40,41], where they found a 2 to 3 times greater probability for an increase in CAD risk in individuals with MS. A positive correlation was observed of CAD risk score and the number of MS components, that is, the greater the number of MS components the higher the risk of developing CAD.

The hyperuricemia, another component related to MS, has been associated with cardiovascular disease and other pathological processes [42]. Within this context, UA has been assessed as an independent risk factor for cardiovascular disease, but results are controversial [43,44]. Our study showed that CAD risk score increases with higher concentrations of UA. The ARIC study [45], in which more than 13,500 individuals, including men and women, participated, did not show any association between hyperuricemia and CAD risk. A recent study [46] conducted in Austria with more than 80,000 men revealed a strong relationship between UA and risk factors for arteriosclerosis. The contradictory results may be justified by methodological differences, such as individuals with a recent history of cardiopathy, use of medications that can influence biochemical results, different ethnic groups or social-economic status [43,45].

One possible, yet contested [47], pathophysiological mechanism of the association between hyperuricemia and CAD could occur by favoring plaque adhesiveness and thus contribute towards atherogenesis and the formation of blood thrombus [48].

A protective effect of muscle mass (MMI) on CAD risk score was found. It is known that the genesis of sarcopenia is associated with an increase in reactive species of oxygen and oxidative stress [49], with a defined role in different types of cardiovascular disease [50]. Weinbrenner et al. [51], studied the relationship between oxidized LDL and other oxidized stress markets with CAD. The authors suggest that the reduction of oxidized LDL, superoxide dismutase and glutathione peroxidase and the increase in oxidized anti-LDL antibodies improves oxidative stress in individuals with CAD.

As in previous studies [52,53], it was possible here to observe the beneficial effects of the recommended intake of SFA and dietary fiber on coronary risk. Jakobsen et al [54] found a positive association between SFA intake and CAD risk among men and women under 60 years of age, but not among individuals over 60 years. In our study the age over 60 seems to influence CAD risk.

A study conducted by Hu et al [55] on types of fat and their relationship to coronary risk underscores the isocaloric replacement of saturated fat with unsaturated fat, which presents a beneficial effect on reducing CAD risk. Our study did not reveal any relationship with mono or polyunsaturated fat. Hu et al. [56] conducted a study with more than 80,000 women between 34 and 59 years of age and found significant relations between CAD risk and types of fat, underscoring the high intake of saturated fat as a risk for CAD [56].

Our study observed that the percentage of energy from protein and the intake of meat are correlated to CAD risk. This is probably due to excess protein of animal origin and the consequent excess intake of saturated fat with higher coronary risks. Individuals with the lower CAD risk score had the lowest legume intake. They also had the lowest caloric intake which could lead to a lower amount of food intake, and a lower intake of legumes.

The mechanism by which SFA influences CAD risk would be by activating Toll-Like 4 receptors, which through free fatty acids stimulate the inflammatory response [57]. Studies have demonstrated the role of inflammation in CAD and other complications [58,59].

The effects of dietary fiber on CAD risk occur through various mechanisms, such as improved lipid standards [60], reduced blood pressure [61] and improved insulin sensitivity [62]. Pereira et al [63] suggest that dietary fiber intake is inversely associated with CAD risk. In this study, the authors indicate a 10 to 30% reduction in coronary risk for every 10g/day of fiber from cereals and fruit [63]. Soluble fiber and its relation to CAD risk has also been shown in several studies [64,65]. Pietinen et al [66] found an inverse association of soluble fiber with CAD risk. In this study, we did not identify the main dietary sources and fiber types and their possible relations with CAD risk score. A review identified 9 prospective studies on the relation of dietary fiber with CAD risk [67]. Among the studies, 7 found a negative association of fiber intake with CAD risk, and 2 studies presented controversial results [67].

In this paper, we studied the role of recommended intake of SFA and dietary fiber on CAD risk score after adjustments for TCV and BMI. The adjustment for total energy intake is used to provide isocaloric conditions for the studied sample [68]. Thus, the existing difference between an individual with TCV of 3000 kcal and another with 1000 kcal would be cancelled, considering only the % of macronutrients. The adjustment for BMI aims at eliminating the effects of adiposity on CAD risk score. Thus, the adjustment for TCV and BMI in the same model aims at annulling excess calories the obese individual may have.

The limitations in this study were the type of study and the food method record. It was a cross-sectional study and some cause/effect relations cannot be proven, only the presence or not of an association between the factors. A single 24-hour dietary recall is based on foods and amounts actually consumed by an individual on one specific day, which has an important limitation for not capture intra-individual variability in food intake. The ideal procedure would be to apply the food method record at least 3 days per week. Furthermore, other limitation was the study sample; a drop-off of 45% could reduce the impact of our results.

Another weak point was related with the muscle mass quantification by BIA in which muscle mass is calculated from lean body mass which is complementary to fat body mass. Then high muscle mass would mean also low fat mass and fat mass is well known as pro-inflammatory and CAD risk factor.

## Conclusion

In this study, recommended intake of saturated fats and dietary fiber are, together with greater muscle mass, inversely associated with CAD risk score. On the other hand, the presence of MS and high plasma uric acid are associated with CAD risk score.

Future proposals for intervention should consider lifestyle changes with proper eating habits and physical exercise (including hypertrophic performance).

## List of abbreviations

BMI: body mass index; CAD: Coronary artery disease; MS: Metabolic Syndrome; γ-GT: γ-glutamyl transferase; WC: Waist circumference; NCEP-ATPIII: National Cholesterol Education Program-Adult Treatment Painel III; HEI: Healthy Eating Index TCV: total caloric value; CRP:C-reactive protein; TC: total cholesterol; n-HDL: non-high density lipoprotein cholesterol; HDL: high density lipoprotein cholesterol; TG: triglycerides. SFA: saturated fatty acid; MUFA: monounsaturated fat acid; PUFA: polyunsaturated fatty acid.

## Competing interests

The authors declare that they have no competing interests.

## Authors' contributions

MMT, ALRC and LASD collected the data and elaborated the manuscript. EPO corrected the manuscript. ALRC and LASD collected the data and wrote the manuscript. FHPB and KCPM revised the final manuscript. RCB was the mentor of the work and advisor of the authors. All authors read and approved the final manuscript.
